# Fetal Brain Damage in Human Fetuses with Congenital Cytomegalovirus Infection: Histological Features and Viral Tropism

**DOI:** 10.1007/s10571-022-01258-9

**Published:** 2022-08-07

**Authors:** Giulia Piccirilli, Liliana Gabrielli, Maria Paola Bonasoni, Angela Chiereghin, Gabriele Turello, Eva Caterina Borgatti, Giuliana Simonazzi, Silvia Felici, Marta Leone, Nunzio Cosimo Mario Salfi, Donatella Santini, Tiziana Lazzarotto

**Affiliations:** 1 Microbiology Unit, IRCCS Azienda Ospedaliero-Universitaria di Bologna, Bologna, Italy; 2 Pathology Unit, Azienda USL-IRCCS di Reggio Emilia, 42123 Reggio Emilia, Italy; 3 Section of Microbiology, Department of Experimental, Diagnostic and Specialty Medicine, University of Bologna, Bologna, Italy; 4 Department of Obstetrics and Gynecology, IRCCS Azienda Ospedaliero-Universitaria di Bologna, Bologna, Italy; 5 Pathology Unit, AUSL della Romagna, Ospedale degli Infermi, Rimini, Italy; 6 Pathology Unit, IRCCS Azienda Ospedaliero-Universitaria di Bologna, Bologna, Italy

**Keywords:** Cytomegalovirus, Brain damage, Congenital infection, Human fetuses, Viral tropism, Neural/neuronal cells

## Abstract

Human cytomegalovirus (HCMV) causes congenital neurological lifelong disabilities. To date, the neuropathogenesis of brain injury related to congenital HCMV (cCMV) infection is poorly understood. This study evaluates the characteristics and pathogenetic mechanisms of encephalic damage in cCMV infection. Ten HCMV-infected human fetuses at 21 weeks of gestation were examined. Specifically, tissues from different brain areas were analyzed by: (i) immunohistochemistry (IHC) to detect HCMV-infected cell distribution, (ii) hematoxylin–eosin staining to evaluate histological damage and (iii) real-time PCR to quantify tissue viral load (HCMV-DNA). The differentiation stage of HCMV-infected neural/neuronal cells was assessed by double IHC to detect simultaneously HCMV-antigens and neural/neuronal markers: nestin (a marker of neural stem/progenitor cells), doublecortin (DCX, marker of cells committed to the neuronal lineage) and neuronal nuclei (NeuN, identifying mature neurons). HCMV-positive cells and viral DNA were found in the brain of 8/10 (80%) fetuses. For these cases, brain damage was classified as mild (*n* = 4, 50%), moderate (*n* = 3, 37.5%) and severe (*n* = 1, 12.5%) based on presence and frequency of pathological findings (necrosis, microglial nodules, microglial activation, astrocytosis, and vascular changes). The highest median HCMV-DNA level was found in the hippocampus (212 copies/5 ng of human DNA [hDNA], range: 10–7,505) as well as the highest mean HCMV-infected cell value (2.9 cells, range: 0–23), followed by that detected in subventricular zone (1.7 cells, range: 0–19). These findings suggested a preferential viral tropism for both neural stem/progenitor cells and neuronal committed cells, residing in these regions, confirmed by the expression of DCX and nestin in 94% and 63.3% of HCMV-positive cells, respectively. NeuN was not found among HCMV-positive cells and was nearly absent in the brain with severe damage, suggesting HCMV does not infect mature neurons and immature neural/neuronal cells do not differentiate into neurons. This could lead to known structural and functional brain defects from cCMV infection.

**Graphical Abstract:**

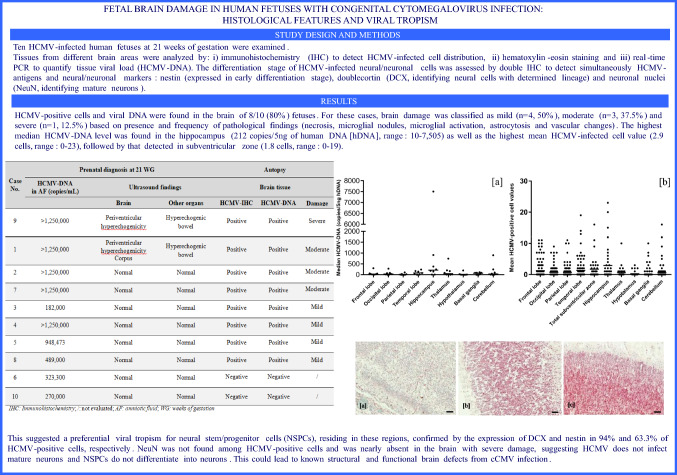

## Introduction

Human cytomegalovirus (HCMV) is the most frequent congenital virus infection worldwide, with an incidence of approximately 0.64% live births (Mack et al. 2017; Rawlinson et al. 2017). Although congenital HCMV (cCMV) infection may involve many fetal organs, the most significant injuries are those related to lesions in the nervous system that are irreversible and persist for life (Fowler et al. 2003; Gabrielli et al. 2009; Kenneson and Cannon 2007; Zhang and Fang 2019). Permanent neurological damage occurs in up to 60–70% of newborns symptomatic at birth (10% of congenitally infected cases) and in the 10–15% of those asymptomatic (Cheeran et al. 2009; Gaytant et al. 2002; Manicklal et al. 2013; Zhang and Fang 2019). Neurological outcomes may include sensorineural hearing loss, visual impairments, microcephaly, encephalic palsy, mental retardation, epilepsy and seizure (Boppana et al. 2013; Cheeran et al. 2009; Gaytant et al. 2002; Schleiss 2008; Zhang and Fang 2019). Clinical conditions involving the central nervous system (CNS) are mainly the result of intracranial calcifications, periventricular cysts, white matter abnormalities, intraventricular adhesions, ventriculomegaly, altered sulcation/gyral patterns and cerebellar abnormalities (Fink et al. 2010; Kwak et al. 2018; Malinger et al. 2003). To date, the pathogenesis of injury in the developing fetal brain related to cCMV infection is poorly understood (Cheeran et al. 2009; Gaytant et al. 2002; Schleiss 2008; Zhang and Fang 2019). Only few studies investigated brain infection and damage in HCMV-infected human fetuses. The authors showed that cerebral injuries could be the result of a multifactorial process including the effects in the brain of direct viral replication, inflammatory and immune response to infection as well as the encephalic hypoxic ischemia secondary to HCMV-related placental damage (Gabrielli et al. 2009, 2012; Sellier et al. 2020; Teissier et al. 2014).

The development of the CNS is a complex process that includes proliferation and differentiation of immature neural/neuronal cells, neuronal migration, neurite outgrowth, and synapse formation (Zhang and Fang 2019). Viral replication could have a key role in neuropathogenesis of HCMV-induced brain injury interfering with these developmental stages. Studies, mainly performed on cultured murine and human brain cells, indicate that CNS infected cells have altered intercellular communication, deficient response to neurotransmitters and cell cycle changes (Cheeran et al. 2009; Han et al. 2017; Ho and van den Pol 2007; Layrolle et al. 2021; Lokensgard et al. 1999; Odeberg et al. 2006, 2007; Rolland et al. 2016; Schleiss 2008; Spector 2015; Zhou et al. 2022). In addition, Tsutsui Y et al. showed a preferential tropism of murine cytomegalovirus (MCMV) for the immature and proliferating neural/neuronal cells (Tsutsui et al. 2002, 2005). In cultured human cells, other studies also confirmed that the neural precursor cells resulted permissive for HCMV infection (Odeberg et al. 2006; Luo et al. 2008, 2010). These findings could also implicate a specific brain localization of viral infection, as well as those described for other herpes viruses, such as the herpes simplex virus-1 (HSV-1), for which temporal lobe abnormalities are commonly reported in patients with herpes simplex encephalitis (Levitz 1998; Mook-Kanamori et al. 2009). All these features were poorly investigated in human cases of cCMV infection.

The present study aimed to implement the knowledge on histological characteristics of HCMV-induced encephalic injury and the mechanism by which the direct viral replication lead to neurological disorders. To our knowledge, this is the first study that analyzed human brain tissues from fetuses at the same gestational age with cCMV infection occurred during the first trimester to define in different encephalic areas the distribution of: (i) histological features, (ii) HCMV-infected cells and (iii) tissue viral load. In addition, the differentiation stage of infected neural/neuronal cells was evaluated to clarify how HCMV infection could interfere with fetal brain development.

## Materials and Methods

### Study Design and Patients

All congenitally HCMV-infected fetuses submitted for histological examination at Pathology Unit, IRCCS S. Orsola Polyclinic from 2010 to 2019 were revised. We selected all fetuses with the same features: mothers with primary HCMV infection occurring before twelve weeks of gestation (WG), high viral load in amniotic fluid, elective termination of pregnancy at 21 WG, all brain regions with available paraffin-embedded blocks. Ten fetuses met these inclusion criteria. In Table 1, maternal clinical information at the time of primary HCMV infection are summarized.

**Table 1:** Maternal clinical information at the time of serological diagnosis of primary HCMV infection

Case no	Age (years)	Serological diagnosis of primary CMV infection	WG at diagnosis	Symptoms	Laboratory findings
9	33	IgG seroconversion	11	–	Lymphocytosis
1	29	IgM antibodies and low IgG avidity	9	–	–
2	25	IgM antibodies and low IgG avidity	12	Influenza-like syndrome	Lymphocytosis and abnormal liver enzyme levels
7	35	IgM antibodies and low IgG avidity	10	Fever	Abnormal liver enzyme levels
3	37	IgM antibodies and low IgG avidity	8	–	–
4	19	IgM antibodies and low IgG avidity	10	Asthenia	Abnormal liver enzyme levels
5	22	IgG seroconversion	11	–	–
8	33	IgM antibodies and low IgG avidity	11	Fever	–
6	32	IgG seroconversion	9	–	–
10	39	IgM antibodies and low IgG avidity	10	–	–

*WG* weeks of gestation, – none; the cases have been ordered like Table 2.

**Table 2:** Results of prenatal diagnosis performed at 21 WG in correlation with histological and virological brain findings

Case no	Prenatal diagnosis at 21 WG			Autopsy			
	HCMV-DNA in AF (copies/mL)	Ultrasound findings		Brain tissue			
		Brain	Other organs	HCMV-IHC	HCMV-DNA	Damage	
9	> 1,250,000	Periventricular hyperechogenicity	Hyperechogenic bowel	Positive	Positive	Severe	
1	> 1,250,000	Periventricular hyperechogenicity Corpus callosum hypoplasia	Hyperechogenic bowel	Positive	Positive	Moderate	
2	> 1,250,000	Normal	Normal	Positive	Positive	Moderate	
7	> 1,250,000	Normal	Normal	Positive	Positive	Moderate	
3	182,000	Normal	Normal	Positive	Positive	Mild	
4	> 1,250,000	Normal	Normal	Positive	Positive	Mild	
5	948,473	Normal	Normal	Positive	Positive	Mild	
8	489,000	Normal	Normal	Positive	Positive	Mild	
6	323,300	Normal	Normal	Negative	Negative	–	
10	270,000	Normal	Normal	Negative	Negative	–	

IHC: Immunohistochemistry; -: not evaluated; AF: amniotic fluid; WG: weeks of gestation; the cases are ordered based on brain damage

As control cases, seven fetuses at 21-weeks of gestation from HCMV seronegative women were also studied. Three were from a termination of pregnancy due to fetal cardiac malformation and the other four were spontaneous miscarriages caused by cervical incompetence. A total of 17 fetuses were analyzed and for each one, 10 brain regions were selected. In particular, tissues from the cortical areas (frontal, temporal, occipital, parietal) and underlying white matter, subventricular zone, thalamus, hypothalamus, hippocampus, basal ganglia and cerebellum were analyzed. All encephalic areas were examined by (i) immunohistochemical staining to detect the presence and distribution of brain cells expressing HCMV-antigens; (ii) hematoxylin–eosin staining to evaluate the histological damage in HCMV-positive brain; and (iii) real-time PCR to detect and quantify the tissue viral load. Double immunohistochemical staining for simultaneous detection of HCMV-antigens with markers identifying the differentiation stage of infected neural/neuronal cells, was performed. All analyses were carried out on sections obtained from paraffin-embedded blocks of tissue previously fixed in 4% formaldehyde.

Regarding histological analysis, a positive and a negative standardized sample were included for each immunohistochemical session in order to confirm the required sensitivity and specificity of the antibodies used. In particular, tissues that did not express the target protein were used to confirm antibody specificity to the protein of interest.

All the histological slides were analyzed by two independent observers. Discordant or dubious results in immunohistochemistry about HCMV-antigen and/or neural/neuronal marker expression were resolved by a third observer. The final decision was determined by at least two observers’ agreement.

The study was approved by the Ethical Committee of St. Orsola Polyclinic, University Hospital, Bologna, Italy (approval numbers: 14/2017/U/tess and 8/2010/O/Sper). The fetal tissues were analyzed after receiving informed consent from the parents prior to inclusion, according to the policies of the Ethical Committee and to the regulation of the Italian Ministry of Health.

### Histological Brain Damage Evaluation in HCMV-Positive Brain

One section of 3 microns/brain area was analyzed by hematoxylin–eosin staining, performed by standard method. The presence of necrosis, microglial nodules, microglial activation, astrocytosis, and vascular changes was evaluated. Microglial nodules consisted in clusters of histiocytes, lymphocytes, and activated microglial cells involved in immune response to HCMV-infected cells. Microglial activation was proved by microglial body cells with various morphologies: small rod-shape, amoeboid-like and spherical cells. Astrocytosis was characterized by cell body expansion (hypertrophy) and cells with clear nuclei (named Alzheimer type II cells). Vascular changes consisted in increased number of vessels with hypertrophy of endothelial cells (that showed plump, cuboidal form and protruded into the lumen).

Considering the severity and the frequency of pathological findings, the brain damage was classified as i) severe, in presence of tissue necrosis and multiple microglial nodules (≥ 3/brain region); ii) moderate, in presence of multiple microglial nodules without necrosis and iii) mild, in presence of occasional microglial nodules (< 3/brain region) without necrosis. In all brain regions, 5 fields at 20 high-power field (HPF), were evaluated (Gabrielli et al. 2012).

### Detection and Quantification of Brain Tissue Viral Load

Each selected brain area was anatomically identified, dissected from 2 sections of 8 microns and placed in a 1.5 mL tube for the deparaffinization procedure using 160 µL of the Deparaffinization Solution (product code: 19093; Qiagen, Germany). DNA extraction was performed by the QIAsymphony^®^ SP instrument with the QIAsymphony DSP DNA Mini Kit (product code: 937236; Qiagen, Germany). Purified DNA was eluted in 50 μL and the contained human DNA (hDNA) was quantified using a real-time PCR assay, Quantifiler^®^ Human DNA Quantification Kit (product code: 4343895; Life Technologies, USA). Five nanograms of hDNA were processed for HCMV-DNA quantification, carried out using a real-time PCR assay, CMV ELITe MGB™ kit (product code: RTK015PLD; ELITech Group, Italy). The tissue viral load was reported as number of copies/5ng of hDNA. The lower limit of quantification (LLoQ) was equal to 13 copies/5 ng of hDNA. Positive results below the LLoQ were censored with a value equal to 10 copies/5ng hDNA (Gabrielli et al. 2017).

Although all brain regions were anatomically identified, the tissue viral load in the subventricular zone was not evaluated due to the difficulties to dissect and scrape the extremely thin layer of the periventricular region.

### Detection and Distribution of HCMV- Infected Brain Cells

One section of 3 microns/brain area was analyzed by immunohistochemical staining, performed using anti-CMV [8B1.2, 1G5.2, 2D4.2] mouse monoclonal primary antibody (product code: 213 M-26; Cell Marque, USA), that reacts with immediate early, delayed early, and late HCMV-antigen preparation. The HCMV-infected cells distribution in the brain was assessed analyzing 5 fields/brain regions at 10 HPF. The results were expressed as mean of HCMV-positive cells/encephalic area (Gabrielli et al. 2019, 2020).

### Differentiation Stage of the HCMV-Infected Neural/Neuronal Cells

Serial sections of 3 microns/brain area were analyzed by double immunohistochemical staining for simultaneously detection of HCMV-antigens with markers of neural/neuronal cells. In particular, nestin was used as a marker of neural stem/progenitor cells, DCX was used to identify cells committed to the neuronal lineage, and NeuN for mature neurons detection (Bernal and Arranz 2018; Bott et al. 2019; Gusel'nikova and Korzhevskiy 2015; Kempermann et al. 2004; Walker et al. 2007; Yin et al. 2013).

The first immunohistochemical reaction was the anti-HCMV staining (pre-diluted mouse monoclonal anti-CMV, clone 8B1.2 1G5.2&2D4.2, Cell Marque USA), visualized using the OptiView DAB detection kit (brown color), followed by the second antibody represented by monoclonal anti-nestin clone 10c2 (product code: SC23927; Santa Cruz Biotech, USA) diluted 1:400, monoclonal anti-DCX clone 2G5 (product code: MABN707; Millipore USA) diluted 1:600 or polyclonal anti-NeuN (product code: ab128886; Abcam Ltd, UK) diluted 1:300. The second immunoreaction was visualized using the Alkaline phosphatase UltraView detection Kit (product code: 760-501; red color). The double-labeled cells were assessed by analyzing 5 fields/brain regions at 10 HPF (Chen et al. 2010).

### Statistical Analysis

The Mann–Whitney test was used to compare the HCMV-DNA levels detected in severe and moderate brain damage *versus* mild brain damage cases. The same test was used to compare the mean values of HCMV-infected cells found in the subventricular zone and in the hippocampus. The differences in HCMV-DNA levels and HCMV-infected cells of each brain regions were analyzed with Kruskal–Wallis test and One-Way ANOVA test, respectively. The mean values of HCMV-infected cells found in severe and moderate brain damage were compared with those detected in mild brain damage cases by independent-test.

## Results

HCMV-positive cells and HCMV-DNA were found in the brain of 8/10 (80%) fetuses. These results together with the data obtained by invasive (detection of HCMV-DNA in amniotic fluid) and non-invasive (ultrasound examination) prenatal diagnosis, performed at 21 WG, are reported in Table 2. For the subsequent examinations, the attention was focused on the cases with an encephalic involvement of HCMV infection. In the remaining 2 fetuses (case number 6 and 10), no HCMV-positive cells and no viral DNA in the brain were observed. In control cases, no HCMV-positive cells and HCMV-DNA were detected in the brain.

### Detection of Pathological Findings and Evaluation of Brain Injury

**Fig. 1:**
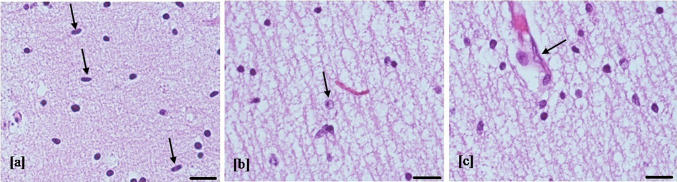
Histological findings in brain injury. Diffuse microglial activation (increased number of rod-shaped cells, arrows) (**a**), astrocytosis (cell with clear nuclei, named Alzheimer type II, arrow) (**b**) and vascular changes (plump endothelial cell protruding into the vessel lumen, arrow) (**c**) detected in all brain regions (hematoxylin–eosin staining 40 HPF, scale bar 50 µm; frontal lobe, case number 3)

**Fig. 2:**
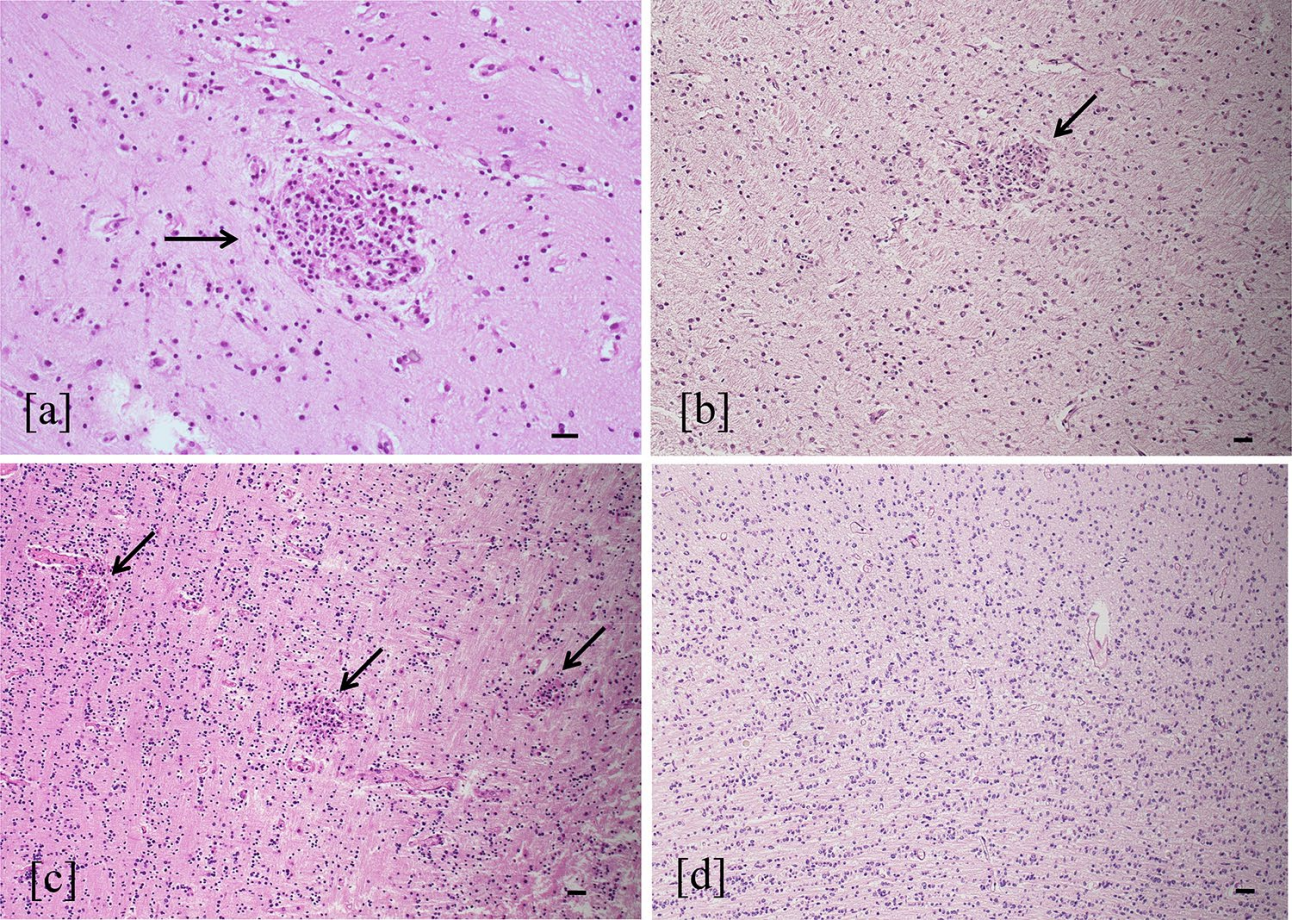
Microglial nodules. Microglial nodules (arrow) composed of histiocytes (grooved reniform nucleus), lymphocytes (round nucleus), and microglial cells (slender nucleus) (**a**) were occasional (< 3/brain region) in mild brain damage (**b**) (hematoxylin–eosin staining, 20 and 10 HPF, scale bar 50 µm; white matter temporal lobe, case number 8). Microglial nodules are multiple (≥ 3/brain region) in moderate and severe brain injury (**c**) (hematoxylin–eosin staining, 10 HPF, scale bar 50 µm; white matter temporal lobe, case number 7). Microglial nodules were absent in control cases (**d**) (hematoxylin–eosin staining, 10 HPF, scale bar 50 µm; white matter temporal lobe)

**Fig. 3:**
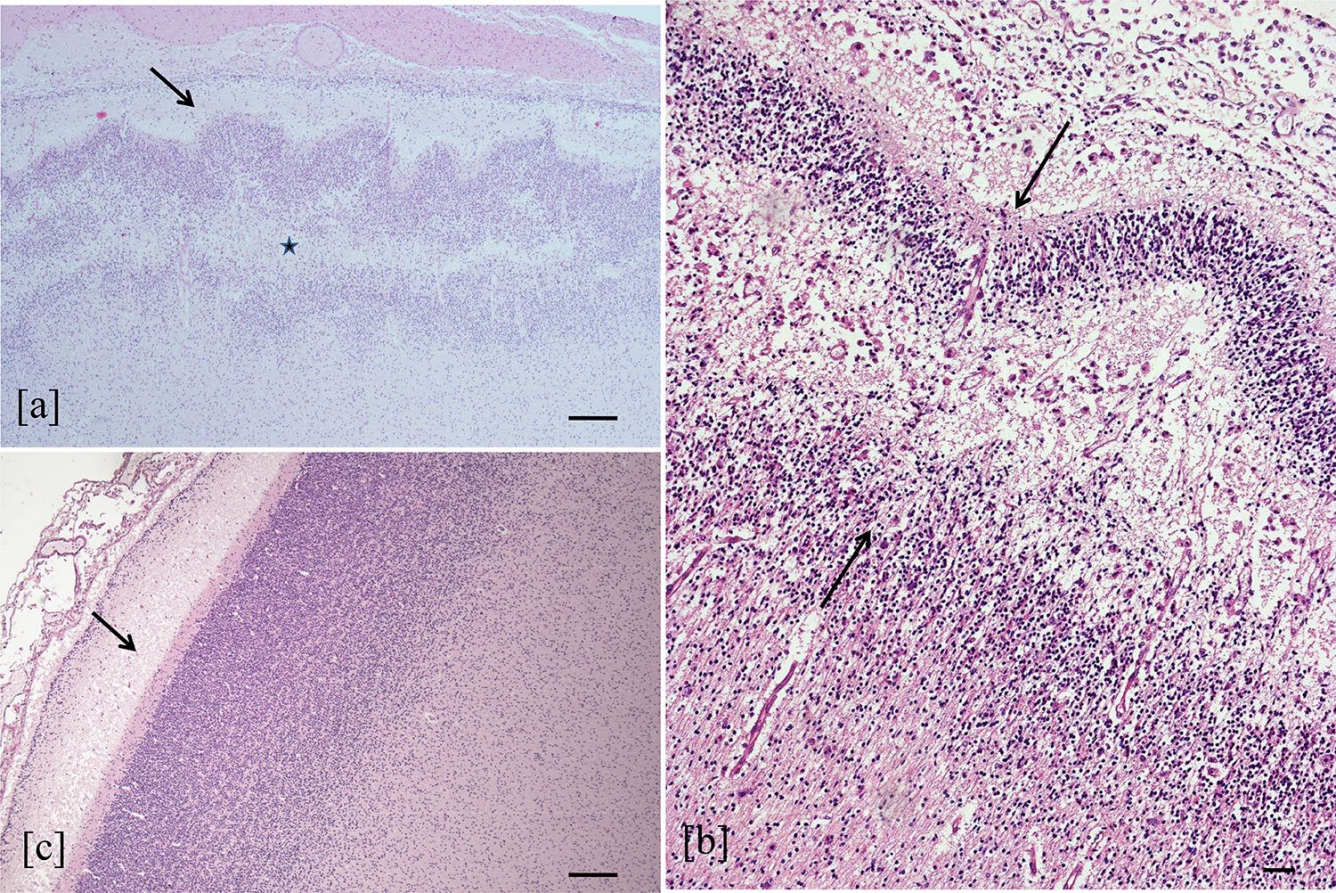
Cortical necrosis and polymicrogyria. Necrosis in cortical layer III (star) and polymicrogyria (arrow) (**a**) were found in the brain with severe injury (hematoxylin–eosin staining, 4 HPF, scale bar 500 µm; parietal lobe, case number 9). Necrosis area at higher magnification (between arrows) (**b**) showed stromal lysis, fewer cortical cells, and numerous macrophages (hematoxylin–eosin staining, 20 HPF, scale bar 100 µm; parietal lobe, case number 9). Polymicrogyria was evident as abundant cortical folds compared to normal cortex with a smooth surface (arrow) and regular neuronal migration (**c**) (hematoxylin–eosin staining, 4 HPF, scale bar 500 µm; parietal lobe, control case)

Microglial activation, diffuse astrocytosis and vascular changes were detected in all 8 cases (Fig. 1), without differences in the brain region distribution. These evidences showed a diffuse encephalic inflammatory reaction. However, as previously reported, considering the severity and the frequency of some histological encephalic abnormalities, such as microglial nodules and necrosis (Figs. 2 and 3), a different degree of brain injury was identified. The brain damage was classified as severe, moderate and mild in 1 (12.5%), 3 (37.5%) and 4 (50%) cases, respectively (Table 2). In particular, the brain with severe damage (case 9) showed cortical necrosis, mainly detected in layer III, diffuse macrophage infiltration of the leptomeninges, polymicrogyria and periventricular leukomalacia. For the same case the cerebellum showed an extensive parenchymal hemorrhage with scarce residual tissue identified. In all cases with severe/moderate encephalic damage, viral load higher than >10^6^ copies/mL in amniotic fluid were found, and the 50% of these cases showed pathological findings involving the brain via ultrasound (Table 2).

### Quantification and Distribution of Tissue Viral Load into the Brain

**Fig. 4:**
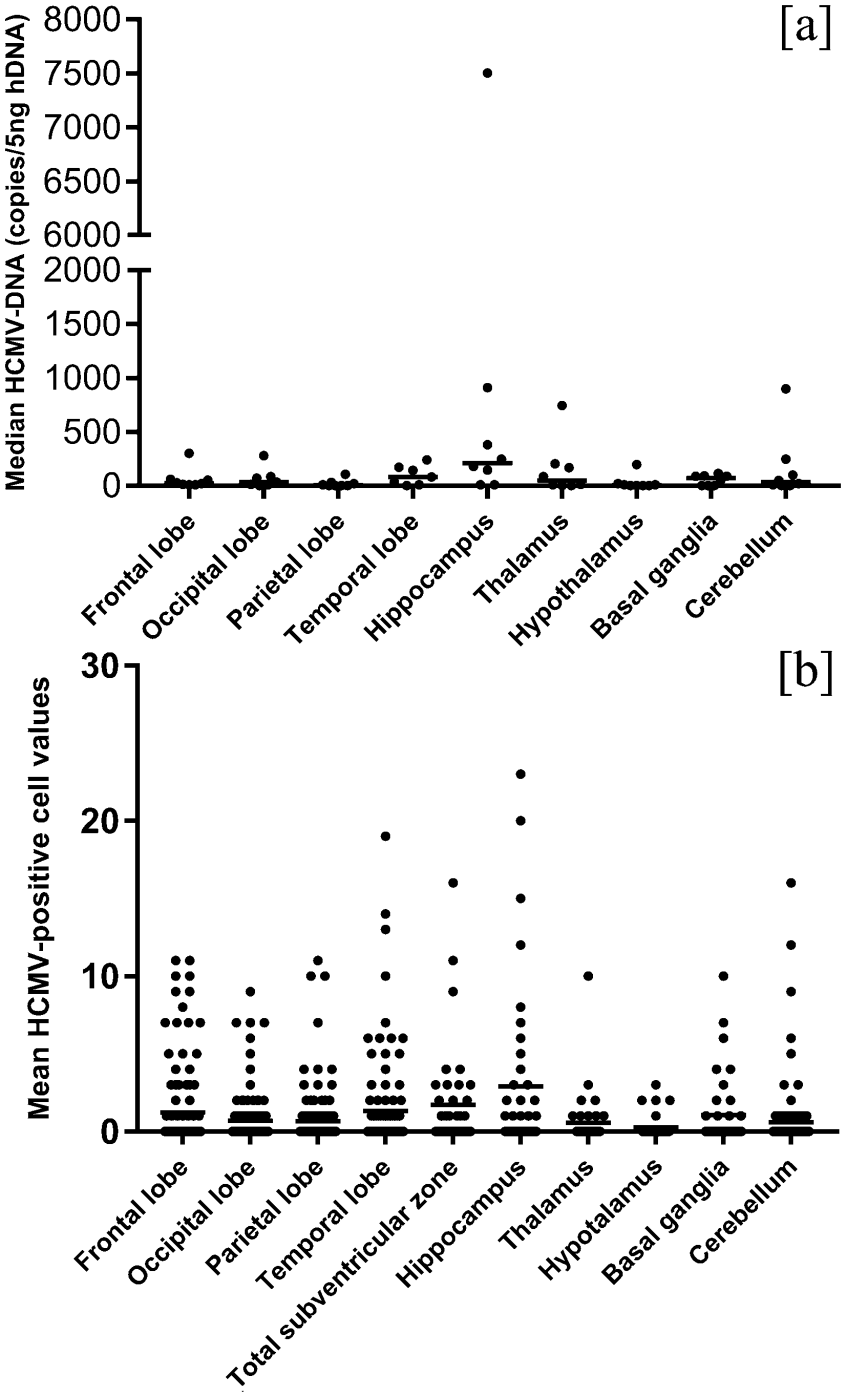
HCMV-DNA levels and HCMV-positive cell distribution in the brain. Tissue viral load (**A**) and distribution of mean HCMV-infected cell values (**B**) in different brain areas. Total subventricular zone refers to both periventricular areas and ganglionic eminence. Mean HCMV-infected cell values on four lobes were calculated considering only the positive cells detected on the cortex and underlying white matter. Tissue viral load in the subventricular zone was not evaluated due to the difficulties to anatomically dissect the thin layer of the periventricular region

The median HCMV-DNA levels detected in the brain of cases with severe and moderate damage were higher than those with mild encephalic injury: 92 copies/5 ng hDNA (range: 20–380 copies/5 ng hDNA) and 87 copies/5 ng hDNA (range: 10–7505 copies/5 ng hDNA) *versus* 10 copies (range: 10–248 copies/5 ng hDNA), respectively (*Z* = 4.827, *p* < 0.0001). The median level of tissue viral load in each brain area was different (*H* = 13.795 *p* = 0.08) and the highest viral load, equal to 212 copies/5ng hDNA (range 10–7505 copies/5 ng hDNA), was detected in the hippocampus (Fig. 4a).

Stratifying the median HCMV-DNA levels in correlation with the degree of brain damage, the highest values were also identified in the hippocampus. Values equal to 380 copies/5 ng hDNA, 910 copies/5 ng hDNA (range 105–7505 copies/5 ng hDNA) and 93 copies/5 ng hDNA (range 10–248 copies/5 ng hDNA) were detected in the cases with severe, moderate and mild brain damage, respectively (data not shown). Tissue viral load in severe brain damage was referred to the only case available (case 9). In addition, in this case, the HCMV-DNA values found in the cerebellum may be biased because it was severely hemorrhagic.

### Quantification and Distribution of HCMV-Positive Cells into the Brain

**Fig. 5:**
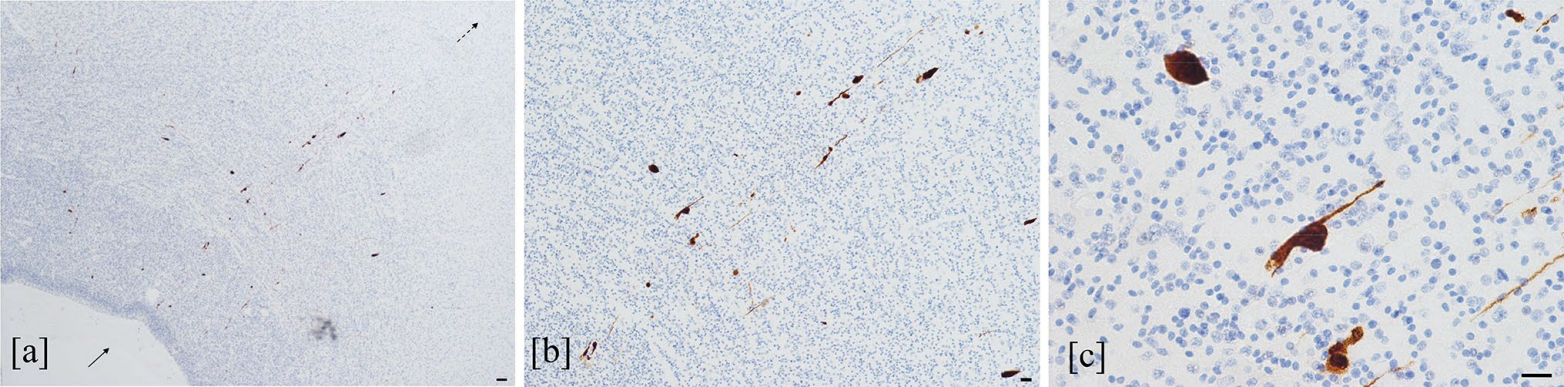
HCMV-infected cells along the migration pathway. HCMV-infected cells (brown-stained cells) from subventricular zone (filled arrow) to cortex (dashed arrow) (**a**). Most of these positive cells, located along the migration pathway, show slender extension typical of radial glia (**b**), as more highlighted in (**c**) (HCMV immunohistochemistry, 4 HPF, scale bar 500 µm; 10 HPF, scale bar 50 µm; 40 HPF scale bar 50 µm; respectively. Temporal lobe, case number 7)

The mean values of HCMV-positive cells, counted in each field over the different brain regions, in the cases with severe and moderate encephalic injury were higher than those found in the fetuses with mild brain damage: 2.49 cells (range: 0–9 cells) and 1.57 cells (range: 0–23 cells) *versus* 0.22 cells (range: 0–11 cells), respectively (*t* = 8.443, *p* < 0.00001). The mean value of HCMV-infected cells in each brain area was different (*F* = 2.311, *p* = 0.02) and the highest value was found in the hippocampus with 2.9 cells (range: 0–23 cells) followed by subventricular zone (including the periventricular areas in each lobe and the ganglionic eminence), with 1.7 cells (range: 0–19 cells) (Fig. 4b). Considering the hippocampus and the subventricular zone only, no statistically significant difference was found between the two areas (*Z* = − 1.064, *p* = 0.224). In addition, the mean values of HCMV-positive cells detected in the germinal matrix were higher than those observed in the cortical area and in white matter (3.5 cells in germinal matrix [range 0–19 cells], 0.8 cells in white matter [range: 0–7 cells] and 0.5 cells in cortex [range: 0–4 cells]). In particular, this was observed in each brain lobe with the highest value in the germinal matrix of temporal lobe (data not shown). On the whole, HCMV-positive cells, including neural/neuronal, glial and endothelial cells were present as both scattered and grouped together in clusters. The HCMV-infected cells were also detected along the migration pathway probably affecting radial glia (Fig. 5).

### Differentiation Stage of the HCMV-Infected Neural/Neuronal Cells

The differentiation stage of HCMV-infected neural/neuronal cells was evaluated in 6 out of the 8 fetuses with HCMV-positive cells in the brain (cases in Table 2: 1, 2, 5, 7, 8, 9).

**Fig. 6:**
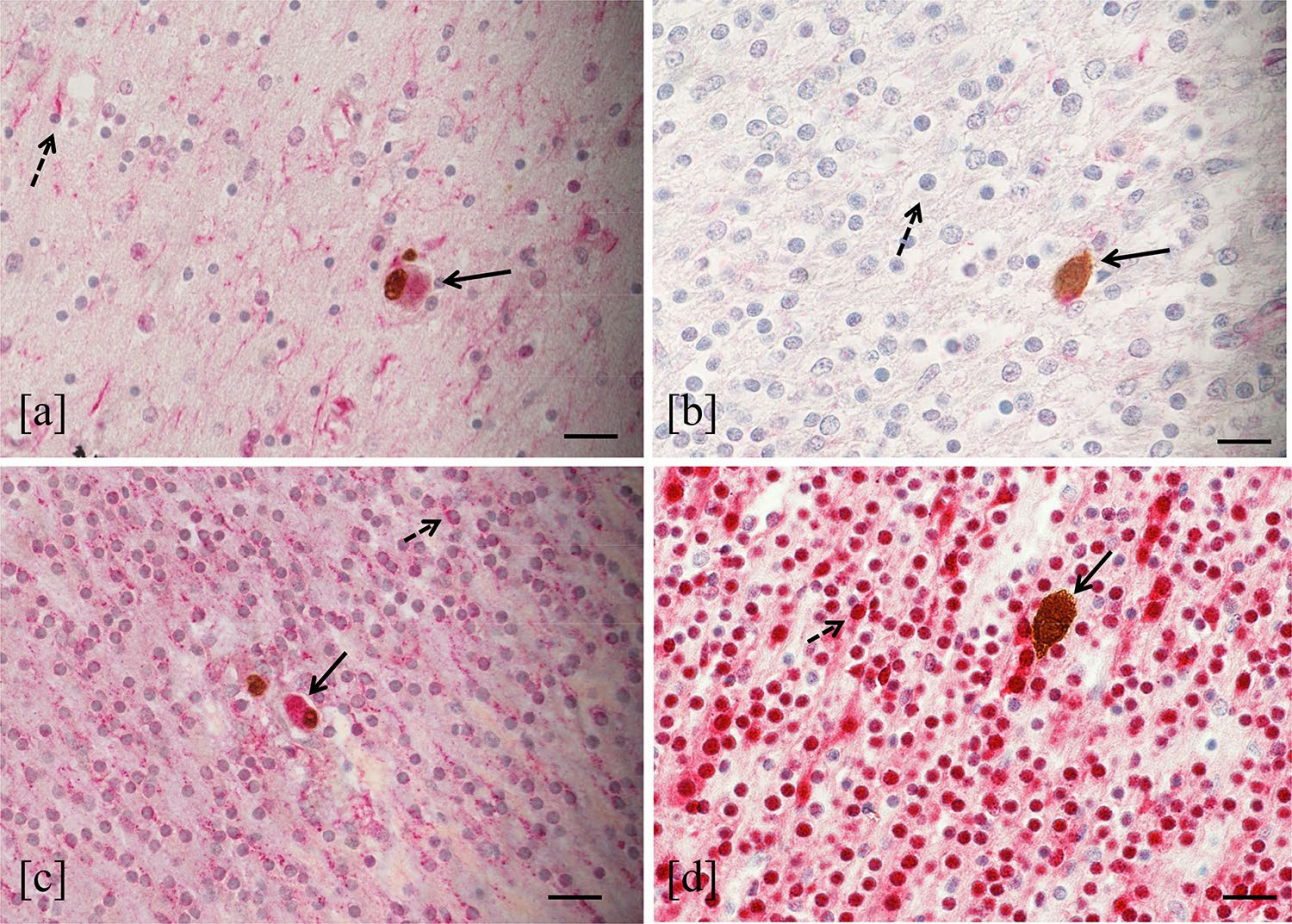
HCMV-antigen and neural/neuronal cell marker expression. HCMV-positive cell expressing nestin identified by both brown and red staining (filled arrow) in a cerebral area where nestin (red staining) was expressed by not infected cells (dashed arrow) (**a**) (double immunohistochemistry, 40 HPF, scale bar 25 µm, hippocampus, case number 7). HCMV-positive cell expressing nestin (filled arrow) in a cerebral area where this marker was almost absent by uninfected cells (dashed arrow) (**b**) (double immunohistochemistry, 40 HPF, scale bar 25 µm, cortex temporal lobe, case number 7). HCMV-positive cell expressing DCX (filled arrow) in a cerebral area where DCX (red staining) was expressed by not infected cells (dashed arrow) (**c**) (double immunohistochemistry, 40 HPF, scale bar 25 µm, cortex temporal lobe, case number 7). HCMV-positive cell not expressing NeuN (brown staining, filled arrow) in the cortex where this marker was expressed by uninfected cells (dashed arrow) (**d**) (double immunohistochemistry, 40 HPF, scale bar 25 µm, cortex temporal lobe, case number 7)

The double immunohistochemical staining for simultaneous detection of HCMV-antigens and neural/neuronal cell markers showed that 63.3% of HCMV-infected cells expressed nestin (441 nestin-HCMV-positive cells/696 HCMV-positive cells) and 94% expressed DCX (601 DCX-HCMV-positive cells/639 HCMV-positive cells). In all cases, nestin was mainly expressed in the subventricular zone and hippocampus (Fig. 6a), while in the white matter and cortex was weakly detected. In thalamus, hypothalamus and basal ganglia, this marker was not expressed. However, the positive cells for both HCMV-antigens and nestin were found in all brain regions, including the area where nestin was not usually expressed by non-infected neural cells (Fig. 6b). Considering the results obtained using DCX, almost all HCMV-positive cells (94%) expressed this marker. In the brain of the fetuses studied, DCX was diffusely expressed in each region, furthermore, positive cells for both HCMV-antigens and DCX were detected in all encephalic areas (Fig. 6c). Finally, no HCMV-positive cells expressing NeuN were found (Fig. 6d). This marker was mainly detected in the cortex, less expressed in white matter, and almost not revealed in the subventricular zone and hippocampus of studied cases.

**Fig. 7:**
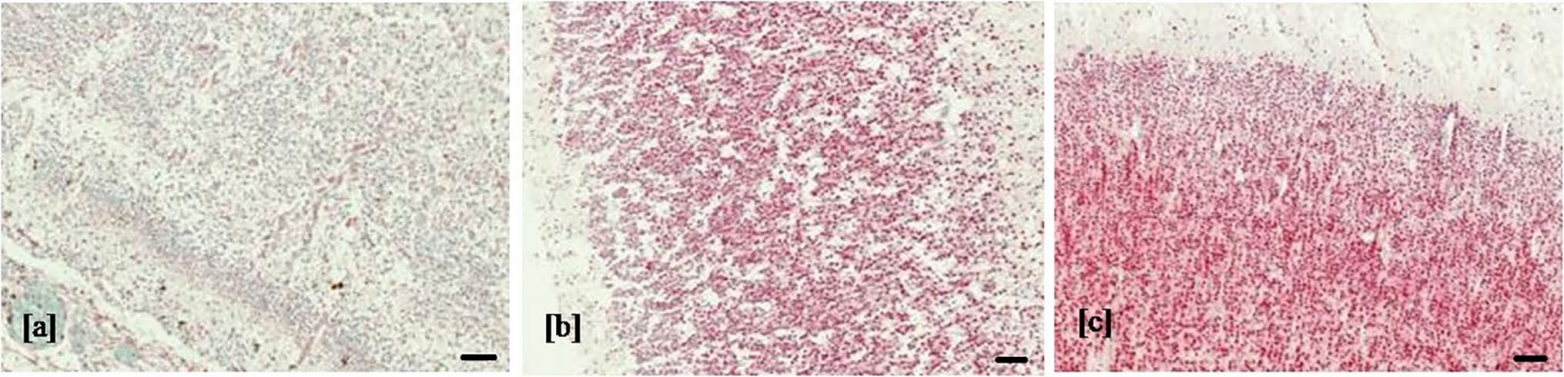
NeuN expression and brain damage. Expression of NeuN (red cells) in cortical area of temporal lobe in case with severe (**a**) moderate (**b**) and mild (**c**) encephalic damage (double immunohistochemistry, 4 HPF, scale bar 500 µm, case number 9, 7, 8)

Considering the same brain region for fetuses with different degree of brain damage, in the case with severe encephalic injury, the expression of NeuN resulted almost absent compared to fetuses with moderate or mild brain damage (Fig. 7).

**Fig. 8:**
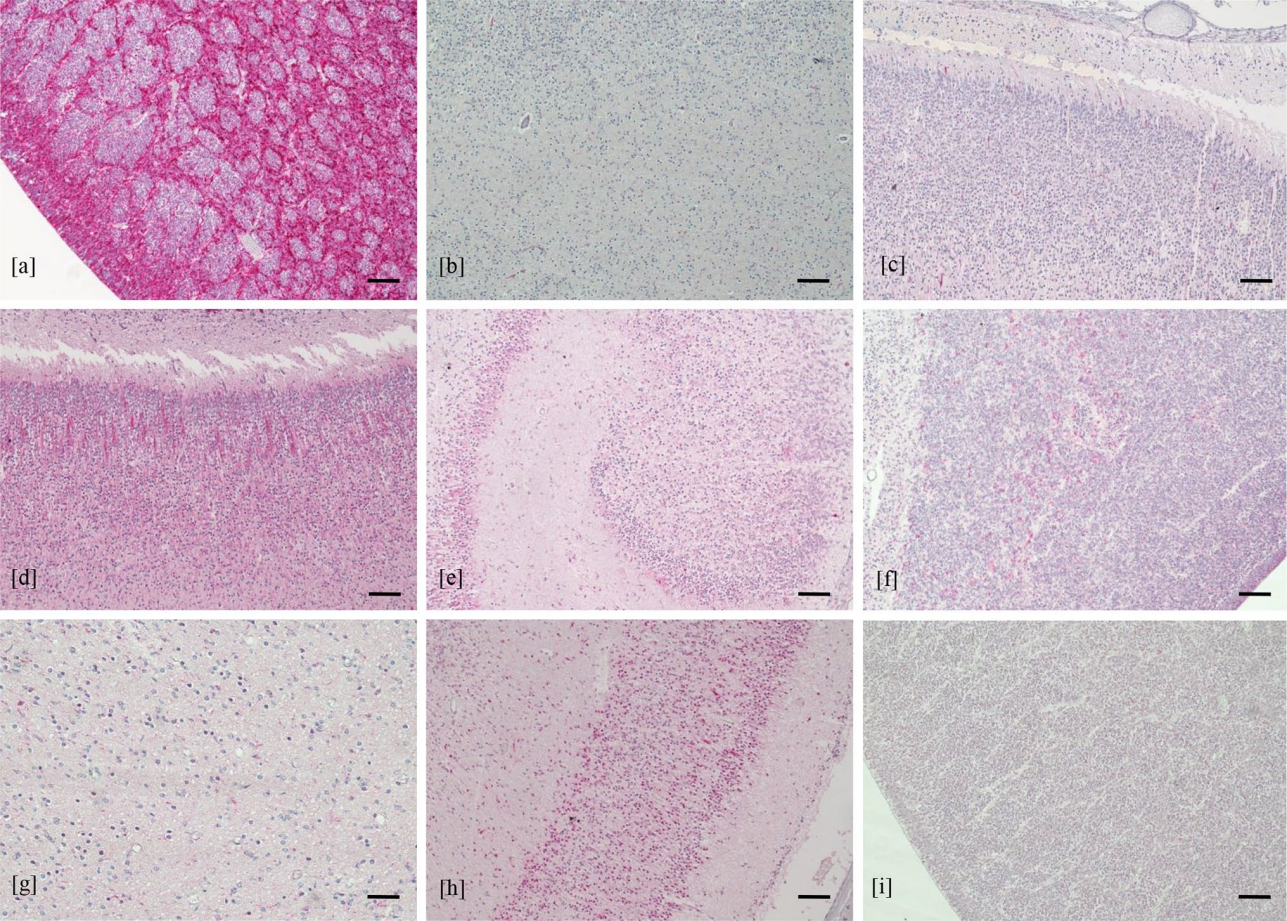
Expression of neural/neuronal cell markers in control cases. Expression of nestin in subventricular zone (**a**), white matter (**b**) and cortical area (**c**); expression of DCX in cortical area (**d**) hippocampus (**e**) subventricular zone (**f**) and white matter (**g**); expression of NeuN in cortical area (**h**) and subventricular zone (**i**) (immunohistochemistry, 4 HPF, scale bar 500 µm)

In the brain of control cases, no pathological findings were found. The nestin was mainly expressed in the hippocampus and the subventricular zone, weakly expressed in the white matter and cortical area and was absent in the thalamus, hypothalamus and basal ganglia (Fig. 8). DCX was diffusely found in all brain regions. NeuN was mainly detected in the cortical area, less expressed in white matter, thalamus and hypothalamus and almost absent in the subventricular zone and in the hippocampus.

## Discussion

Although cCMV infection is the leading cause of significant damage in brain development, little is known about the neuropathogenic mechanisms by which this viral infection leads to human fetal cerebral injury (Cheeran et al. 2009; Gaytant et al. 2002; Schleiss 2008; Zhang and Fang 2019). The results obtained by this study show encephalic viral infection in 80% (8/10) of examined fetuses, proved by the detection of HCMV-positive cells and HCMV-DNA in the brain. This could suggest that not all HCMV-infected fetuses show cerebral involvement of viral replication, as reported in previous studies (Gabrielli et al. 2009, 2012, 2013), however, our data reflected what happened up to 21 WG.

We focused on the 8 cases with HCMV-positive cells and HCMV-DNA detected in the brain. In particular, the HCMV-positive cells were scattered and/or grouped together in clusters, in all the histological sections analyzed. Moreover, the distribution of HCMV-positive cells was not uniform in different fields of the same cerebral region. Many factors can contribute to this finding, for example, the stage of cell differentiation and replication, the immune response, and the dissemination pathway of HCMV infection in the brain (Kamte et al. 2021; Krstanović et al. 2021).

In all HCMV-positive brains, the presence of diffused microglial activation, astrocytosis, and vascular changes observed in all brain regions, suggest a disseminate inflammatory response (Cotran and Pober 1989; Fernández-Arjona et al. 2017; Robillard et al. 2016). In addition, microglial nodules in the same areas analyzed were also found. These lesions are a neuropathological characteristic of viral CNS infections, especially encephalitis caused by HSV-1 and HCMV; being the result of microglial cells involved in phagocytosis of infected brain cells killed by CD8+ cytotoxic T-lymphocytes (Chen et al. 2019; Langford et al. 2002; Ludlow et al. 2016; Nebuloni et al. 2000; Rock et al. 2004; Tröscher et al. 2019). In a previous study, characterizing the encephalic inflammatory infiltrate in human fetuses with cCMV infection, the presence of microglial nodules containing HCMV-positive cells surrounded by activated CD8+ T-cells was demonstrated (Gabrielli et al. 2012). In our study, except for necrosis, all pathological findings were not localized in specific brain areas, but uniformly distributed, probably due to diffuse inflammatory reaction and immune responses into the encephalon. The necrosis, found in the brain with severe damage and mainly localized in cortical layer III, could be due not only to direct viral replication, but also to hypoxia caused by HCMV-induced placental injury (Gabrielli et al. 2012). In the studied cases, the different degree of encephalic injury was classified as severe (12.5%), moderate (37.5%) and mild (50%). In cases with severe/moderate brain damage, prenatal diagnosis performed at 21 WG, showed a high viral load in amniotic fluid (values more than 10^6^ copies/mL) and in 50% of cases, brain pathological features detected by ultrasound examination. These findings are in agreement with published data reporting that the presence of high viral loads in amniotic fluid, sampled at the appropriate time, and only when combined with ultrasound evidence of abnormalities in the CNS, are highly suggestive of fetal cCMV infection with poor outcome (Guerra et al. 2008; Lazzarotto et al. 2014; Pass and Arav-Boger 2018). In addition, in cases with severe/moderate encephalic damage, higher median HCMV-DNA values and mean values of HCMV-positive cells were found compared to those detected in cases with mild brain injury. This confirms a correlation between the degree of damage and the level of viral replication, as already reported (Gabrielli et al. 2012). Compared to other studies in which the Authors investigated fetuses with evident cerebral abnormalities at ultrasound (Teissier et al 2014; Sellier et al. 2020), we specifically wanted to include infected fetuses without ultrasound pathological findings in order to understand better what happened in these cases. We demonstrated the presence of HCMV even in the latter cases that also showed moderate/mild brain damage.

Among brain areas, the highest viral load was identified in the hippocampus (median value 212 copies/5 ng hDNA, range: 10–7505 copies/5 ng hDNA) as well as the highest HCMV-infected cells (mean value 2.9 cells, range: 0–23 cells), followed by subventricular zone (1.7 cells, range: 0–19). In addition, considering each cerebral lobe, the highest mean value of HCMV-positive cells was detected in the germinal matrix (part of subventricular zone) with respect to cortex and white matter. In acute encephalitis due to other neurotropic viruses, such as HSV-1, involvement of select brain regions are reported, including the limbic structure where the hippocampus is a susceptible target of infection (Damasio and Van Hoesen 1985; Harris and Harris 2018; Kopp et al. 2009; Lathe and Haas 2017; Potel et al. 2003). For HCMV infection, the main viral localization in the hippocampus and cerebral subventricular zone was proven in few studies involving human subjects, such as premature infants with lethal cCMV infection and adult immunocompromised patients (Perlman and Argyle 1992; Yoonet al. 2017). In particular, in the latter, Yoon et al. demonstrated a preferential HCMV tropism for the dentate gyrus of the hippocampus (Yoon et al. 2017). In this area neurogenesis is known to persist throughout adult life (Ihunwo et al. 2022).

Sellier et al. also found a preferential localization of HCMV-positive cells in the periventricular and germinative areas (Sellier et al. 2020). Although our results showed a higher mean values of HCMV-infected cells in the hippocampus compared to the subventricular zone, no significant statistically difference was observed. Therefore, further investigations on a bigger population of HCMV fetuses of the same WG should be required to better define if between these two areas there was one more affected by viral replication. Interestingly, Yin et al. observed that during human fetal life, the highest proportion of neural stem/progenitor cells reside in the hippocampus, followed by the amount of these cells detected in subventricular zone (Yin et al. 2013). Considering these findings, though identified in a low number of fetuses studied, the highest viral load detected in hippocampus and the higher HCMV-infected cell values found in both hippocampus and in subventricular zone could probably suggest a preferential HCMV replication in the cerebral areas where immature neural/neuronal cells reside. Murine models of MCMV infection, showed that the immature neural cells were the main target of viral replication (Mutnal et al. 2011; Tsutsui et al. 2002). Moreover, studies performed on human neural precursor cell cultures, demonstrated the greatest susceptibility to HCMV of these cells, in which the effects of viral replication depend on their differentiation state (González-Sánchez et al. 2015; Li et al. 2009; Odeberg et al. 2006). In order to explore these findings, the differentiation stage of HCMV-infected neural/neuronal cells was investigated. In our small population, the main neural/neuronal marker expressed by HCMV-positive cells was DCX (94%), the antigen identifying the neural progenitor cells with a determined lineage (as neuroblasts). Nestin was found in the 63.3% of HCMV-positive cells, while no HCMV-infected cell resulted positive for NeuN. These findings showed that the HCMV-infected cells were mainly neural stem/progenitor cells and neuronal committed cells, also confirming the preferential tropism of HCMV for immature and proliferating cells of neuronal lineage as described by Teissier et al. (Teissier et al. 2014). Further studies on cellular proliferation markers could provide additional information to our results. On the other hand, the total absence of HCMV-infected cells expressing NeuN, could be explained by previous human cell and animal model studies, which showed that mature neurons are refractory to HCMV replication (Cheeran et al. 2009; Cosset et al. 2015; Slavuljica et al. 2015). The HCMV-positive cells that did not express neural/neuronal markers, were probably cells different from those of neuronal lineage. This is in agreement with literature, which reports the ability of HCMV to infect different types of cells in the brain, such as endothelium and glia (Gabrielli et al. 2012). Different hypothesis could explain the high proportion of HCMV-positive cells expressing DCX. The first is that it could reflect the diffuse expression of this marker in all brain regions at 21 WG, as observed in both control cases and published data (Qin et al. 2000); DCX expression gradually decreased there after (Qin et al. 2000). Secondly, as suggested by other Authors, HCMV infection may affect host gene expression including *DCX*. In particular, increased levels of DCX was found in both HCMV-infected neural stem cells and histological sections from fetuses with polymicrogyria/lissencephaly by contrast with uninfected controls (Rolland et al. 2016, 2021). However, compared to our population of fetuses at 21 WG, these cases were at 23 WG and all presented severe brain damage. Instead, in our study the high DCX expression was also found in cases with mild encephalic injury as in controls. Thirdly, it could also be speculated that the massive DCX expression among the HCMV-positive cells may be due to a preferential viral replication in neuronal precursor cells rather than in neural stem cells. In order to verify this hypothesis, further investigations on more human fetal cerebral samples would be required. Finally, the high HCMV-positive cells expressing DCX could be due to an altered ability of infected neuronal progenitor cells to differentiate into mature neurons. The total absence of HCMV-positive cells expressing NeuN, in the cases studied, may be aligned with the latter hypothesis. Studies performed on human cell cultures demonstrated that the HCMV infection in neural precursors led to either the inhibition or delay of differentiation in these cells (Cheeran et al. 2009; Luo et al. 2010; Li et al. 2009; Brown et al. 2019; D'Aiuto et al. 2012; Cosset et al. 2015; Berger et al. 2015). The detection of HCMV-infected cells expressing nestin in all brain regions, also in areas where this marker was scarcely present, may suggest that HCMV infection could interfere with neuronal differentiation. In control cases, as reported in literature, NeuN is mostly expressed in the cortex, weakly in the white matter, and almost absent in the hippocampus (Sarnat et al. 1998). In the latter this finding was probably due to a lower number of maturing neurons. On the other hand, NeuN cortical expression in the only fetus with severe brain damage was almost absent, but preserved in fetuses with moderate/mild injury. Of note, NeuN immunohistochemistry can individuate signs of neuronal suffering; in fact immunoreactivity has been reported to decrease under several pathologic conditions such as fetal hypoxia and maternal smoking (Lavezzi et al. 2013).

The presence of HCMV-infected cells in the pathways of neuronal migration, may suggest that these cells could retain their ability to migrate, although may be aberrant, as reported by other studies (Cheeran et al. 2009; Rolland et al. 2021; Han et al. 2017; Zhou et al. 2022). Moreover, because HCMV-infected cells were found in all subventricular zone, including periventricular region and ganglionic eminence, where immature cells depart for radially and tangentially neuronal migration, respectively, both migration modes could be affected. The neuronal migration process could also be impaired by infected glial cells that we morphologically identified in our cases.

In conclusion, although in a small number of cCMV infected fetuses, this study suggests a preferential viral tropism for neural stem/progenitor cells and neuronal committed cells, that may lose or delay their capacity to differentiate, while may retain their ability to migrate. These mechanisms could lead to abnormal brain development; in fact, it is well known that polymicrogyria and lissencephaly are the result of abnormal neuronal migration (Stouffer et al. 2016; Guerrini and Parrini 2010; Rolland et al. 2021), instead intracranial calcification is indicative of neuronal cell death (Ghidini et al. 1989). All of these features are usually described in infants with HCMV infection (Cheeran et al. 2009; White et al. 2014). The pathological findings detected in this study showed brain features at 21 weeks of gestation and it is difficult to establish the subsequent evolution of the injury, above all in the cases with mild brain damage. The fetuses with severe/moderate cerebral injuries could probably have had a poor outcome, since necrosis, periventricular leukomalacia, polymicrogyria, and multiple microglial nodules are parenchymal brain lesions associated with permanent neurological sequelae (Krstanovic et al. 2021; Leruez-Ville et al. 2020).

In light of these results suggesting the direct role of HCMV in the pathogenesis of cCMV-related brain damage, additional features of cerebral injury will have to be investigated in all encephalic regions, such as the effects of the immune responses and the development degree of additional infected cell types involved in the neurogenesis, such as glial cells.

## Data Availability

Data and materials are available with conditions for access, if the request is reasonable and justified.

## Abbreviations

HCMV: Human cytomegalovirus
cCMV: Congenital HCMV
IHC: Immunohistochemistry
DCX: Doublecortin
NeuN: Neuronal nuclei
hDNA: Human DNA
CNS: Central nervous system
MCMV: Murine cytomegalovirus
HSV-1: Herpes simplex virus-1
WG: Week of gestation
LLoQ: Lower limit of quantification
AF: Amniotic fluid
